# Inactivated pandemic 2009 H1N1 influenza A virus human vaccines have different efficacy after homologous challenge in the ferret model

**DOI:** 10.1111/irv.12784

**Published:** 2020-08-11

**Authors:** Beatriz Vidaña, Sharon M. Brookes, Helen E. Everett, Fanny Garcon, Alejandro Nuñez, Othmar Engelhardt, Diane Major, Katja Hoschler, Ian H. Brown, Maria Zambon

**Affiliations:** ^1^ Bristol Veterinary School Faculty of Health Science University of Bristol Bristol UK; ^2^ Pathology Department Animal and Plant Health Agency APHA‐Weybridge Addlestone UK; ^3^ Virology Department Animal and Plant Health Agency APHA‐Weybridge Addlestone UK; ^4^ Laboratoires Théa Clermont‐Ferrand France; ^5^ National Institute for Biological Standards and Control Potters Bar UK; ^6^ Public Health England – Colindale London UK

**Keywords:** adjuvant, efficacy, ferret, immunopathology, influenza A, pandemic 2009 H1N1, vaccine

## Abstract

**Background:**

The 2009 pandemic H1N1 (A(H1N1)pdm09) influenza A virus (IAV) has replaced the previous seasonal H1N1 strain in humans and continues to circulate worldwide. The comparative performance of inactivated A(H1N1)pdm09 influenza vaccines remains of considerable interest. The objective of this study was to evaluate the efficacy of two licensed A(H1N1)pdm09 inactivated vaccines (AS03B adjuvanted split virion *Pandemrix* from GlaxoSmithKline and referred here as (V1) and non‐adjuvanted whole virion *Celvapan* from Baxter and referred here as (V2)) in ferrets as a pre‐clinical model for human disease intervention.

**Methods:**

Naïve ferrets were divided into two groups (V1 and V2) and immunised intramuscularly with two different A/California/07/2009‐derived inactivated vaccines, V1 administered in a single dose and V2 administered in 2 doses separated by 21 days. Six weeks after the first immunisation, vaccinated animals and a non‐vaccinated control (NVC) group were intra‐nasally challenged with 10^6.5^ TCID_50_ of the isolate A/England/195/2009 A(H1N1)pdm09 with 99.1% amino acid identity to the vaccine strain. Clinical signs, lung histopathology, viral quantification and antibody responses were evaluated.

**Results and Conclusions:**

Results revealed important qualitative differences in the performance of both inactivated vaccines in relation to protection against challenge with a comparable virus in a naive animal (ferret) model of human disease. Vaccine V1 limited and controlled viral shedding and reduced lower respiratory tract infection. In contrast, vaccine V2 did not control infection and animals showed sustained viral shedding and delayed lower respiratory infection, resulting in pulmonary lesions, suggesting lower efficacy of V2 vaccine.

## 1 INTRODUCTION

Since its emergence, the A(H1N1)pdm09 influenza A virus (IAV) has become established worldwide in the human population and replaced the previous seasonal human H1N1 viruses.^1^ Head‐to‐head comparisons of the extent to which diverse influenza vaccines prevent viral infection and disease in biological model systems are infrequent, but important to generate evidence relevant to, reported variations of effectiveness between influenza vaccines deployed in the field.^2^, ^3^ Three vaccine platforms are currently licensed for influenza prevention in humans: inactivated subunit or whole virion vaccines, live attenuated and recombinant haemagglutinin (HA) protein. For inactivated IAV vaccines, reference strains are produced with the HA and neuraminidase (NA) genes derived from the most recently circulating strains, as recommended by the World Health Organization (WHO) Influenza Virus Vaccine Selection Committee, and the internal protein genes from a laboratory influenza strain adapted to grow in eggs.^4^ Inactivated vaccines primarily induce serum antibodies against the HA, which are capable of strain‐specific neutralisation of influenza viral particles thus protecting from infection. In order to increase the production of these antibodies, some licensed inactivated vaccines also include adjuvant, such as AS03 or MF59,^5^ which enhance immunity against the target antigen.

This study evaluates the efficacy of two A(H1N1)pdm09‐derived inactivated vaccines, one adjuvanted *Pandemrix* (AS03B adjuvanted split virion vaccine) referred hereafter as V1 and one non‐adjuvanted *Celvapan* (non‐adjuvanted whole virion vaccine) referred hereafter as V2 at the human dose and schedule^6^ in the ferret (*Mustela putorius furo*) model after challenge with a A(H1N1)pdm09 strain. Vaccines used in this study were selected and compared as part of pandemic vaccination stockpiling policy in preparedness for subsequent influenza pandemic waves. The rationale of the study was to provide comparative data on the immunogenicity and protection in animal models after viral challenge using the human vaccination schedule in clinical trials. The aim of the study was to evaluate vaccine individual efficacy against a single homologous strain challenge as opposed to the efficacy conferred by seasonal multivalent vaccine immunogens, comparing the parameters of clinical signs, viral shedding, histopathology and viral antigen distribution, viral tissue load and serology relating these data to performance of these vaccines in humans.

## 2 MATERIALS AND METHODS

### 2.1 Virus isolation and propagation

The challenge strain was A/England/195/2009 (E195, GQ166661) and hereafter referred to as (A/Eng) A(H1N1)pdm09. The virus was isolated by Madin–Darby Canine Kidney (MDCK) cell culture, plaque purified three times and verified by whole genome sequencing.^7^

### 2.2 Vaccines

Both vaccines used in this study were based on the prototype A(H1N1)pdm09 strain A/California/07/2009 (A/Cal). Vaccine 1 (V1) was a split inactivated influenza virus vaccine constructed from the v‐like strain antigen (New York Medical College x‐179A), generated by classical re‐assortment in embryonated eggs consisting of a 0.25 mL suspension containing 3.75 µg of HA mixed with 0.25 mL emulsion of AS03B adjuvant and the latter composed of squalene (10.69 mg), DL‐α‐tocopherol (11.86 mg) and polysorbate 80 (4.86 mg). Vaccine 2 (V2) consisted of a whole virion influenza vaccine, containing 7.5 µg inactivated HA of A/Cal virus propagated in Vero cells.

### 2.3 Ferret immunisation

Animal experiments were approved by the APHA ethics committee and conform to the ARRIVE guidelines and UK legislation under the Animals (Scientific Procedures) Act 1986 Amendment Regulations (SI 2012/3039). Forty‐three naïve male ferrets of approximately 8 months of age were selected from a stable, purposely bred colony and received a subcutaneous Biothermal microchip (digital angel) implant for identification and temperature monitoring. Ferrets were shown to be seronegative for IAV antibodies using haemagglutination inhibition (HI) assay to the temporally relevant circulating seasonal human vaccine strains: A/Brisbane/59/2007 (H1N1), A/Uruguay/716/2007 (H3N2), B/Brisbane/60/2008 (B/ Victoria lineage), A/England/195/2009, and A/California/7/2009 (A(H1N1)pdm09) and pan‐influenza A ELISA. The absence of influenza A infection was confirmed by verifying that no influenza A virus RNA could be detected in nasal wash samples.

**FIGURE 1:**
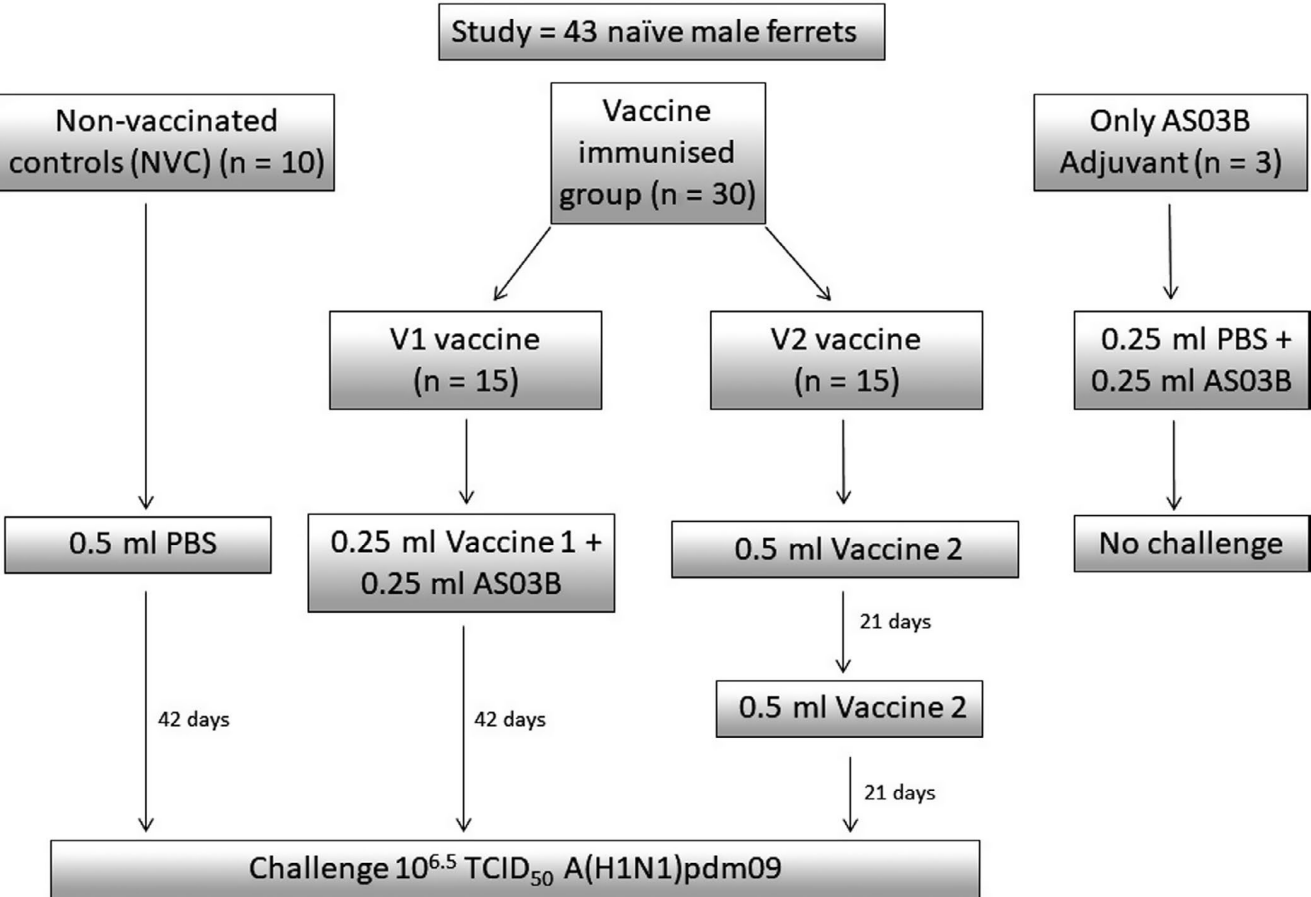
Experimental immunisation and challenge diagram. Ferrets were divided into groups and were either not vaccinated (NVC, n = 10) or were vaccinated with vaccines V1 or V2 (n = 15 for each vaccine) before being challenged. A further groups of 3 ferrets received AS03 adjuvant alone

Ferrets were randomly assigned to different experimental groups (Figure 1). Two experimental groups of 15 animals (vaccine immunised) and one non‐vaccinated control group of 10 animals (NVC) were challenged with 10^6.5^ TCID50 A/Eng/195 grown in MDCK cell culture. Animals in group V1 were immunised with vaccine 1 (0.25 mL of antigen and 0.25 mL of the AS03B adjuvant, mixed immediately before immunisation) in a single dose. The second vaccinated group, V2, was immunised twice with 0.5 mL of the vaccine 2, with a 21‐day interval, mimicking human schedule. The NVC group received a single dose of 0.5 mL of PBS. A fourth group of three animals received AS03B adjuvant only (0.25 mL AS03B mixed with 0.25 mL PBS). All inoculates were administered intramuscularly in the left hind limb. Blood samples were taken under anaesthesia on 0, 7, 11, 14, 18, 21, 28 and 34 days post‐vaccination (dpv).

### 2.4 Ferret experimental infection and clinical signs

Six weeks after the initial immunisation, animals in all groups were challenged with 10^6.5^ TCID_50_ of A/Eng virus in a total of 0.4 mL (0.2 mL per nostril). Ferrets were monitored daily for changes in temperature and body weight. Subsets of animals (5 per group) were humanely killed on 3, 5 and 14 days post‐infection (dpi) by intravenous injection of sodium pentobarbital overdose injection, under anaesthesia with ketamine and medetomidine, and a series of tissues (brain, olfactory bulb, thoracic trachea, nasal turbinate and lung) collected. On alternate days post‐challenge (1‐14), nasal washes and rectal swabs were also collected under anaesthesia. All samples were assayed for the detection of influenza A M gene using specific RT‐qPCR.^8^ Nasal turbinate, trachea and lung tissues were collected for histopathology. Serum antibody responses were evaluated by haemagglutination inhibition (HI) assays at −8 dpi/34 dpv, and at 3, 5 and 14 dpi as previously described.^9^

### 2.5 Histopathology

Samples of nasal turbinate, trachea and each of the right lung lobes were collected from ferrets humanely killed at 3 and 5 dpi and fixed in 10% neutral‐buffered formalin for a minimum of 5 days. During postmortem examination, nasal turbinate samples were collected by carefully removing the nasal concha from the ethmoid and maxillary bone and the nasal septum with a thin scalpel (ie number 11 surgical scalpel blade) after sagittal sectioning of the cranium; no decalcification methods were used. Tissues were routinely processed and embedded in paraffin wax, sectioned at 4 µm, and stained with haematoxylin and eosin (HE) for examination under light microscopy by a veterinary pathologist. In addition, a semi‐quantitative assessment of IAV‐associated microscopic lesions in the lungs was performed separately in cross sections of the right cranial, middle and caudal pulmonary lobes for each animal. The HE lesion scoring was graded on the basis of severity as previously described.^10^ The mean count for all three lobes was used for the final histopathological score for each animal.

### 2.6 Viral detection and quantification

#### 2.6.1 RT‐qPCR

Viral RNA from nasal washes, rectal swabs and tissue homogenates was extracted according to published methods.^8^ The A(H1N1)pdm09 viral matrix gene RT‐PCR was performed as described previously.^11^ *C*_t_ values obtained from clinical specimens were converted to relative equivalent units (REUs) by correlation with a standard line produced using a 10‐fold dilution series of RNA prepared from the same stock as the inoculum with known titre.

#### 2.6.2 Immunohistochemistry (IHC)

Detection of IAV nucleoprotein (NP) by IHC was performed as described^12^ on nasal turbinates, trachea and lungs. Briefly, sections for IHC were dewaxed and endogenous peroxidase activity was quenched with a methanol/hydrogen peroxide block for 15 minutes and treated with Protease XXIV for 10 minutes at room temperature. Primary antibody cross‐reactivity with tissue constituents was prevented using a normal immune serum block. Samples were incubated with an anti‐Influenza A nucleoprotein primary mouse monoclonal antibody (Statens Serum Institut) for 1 hour and Dako ENVISION™ polymer for 30 minutes at room temperature. The immunohistochemical signal was visualised using 3,3‐diaminobenzidine (Sigma‐Aldrich) and counterstained in Mayer's haematoxylin (Surgipath). Viral NP antigen was assessed in all tissues, and levels were semi‐quantitatively evaluated in the lungs using a modified method.^10^, ^13^ Alveolar and bronchial/bronchiolar NP‐positive cell counts were calculated separately. The mean of the total scores for both bronchial parameters across two tissue sections of each lung lobe was calculated for each animal.

### 2.7 Serological analysis

Ferret sera were assayed using A(H1N1)pdm09 influenza A antigens and turkey red blood cells (RBC).^9^ Longitudinal experimental serum samples were analysed during the study by HI using the homologous A/England/195/2009 strain and pan‐influenza A ELISA in order to assess vaccine immunogenicity post‐vaccination and to assess recall response post‐challenge.

### 2.8 Statistical analysis

Graphics and statistical differences between groups were analysed using GraphPad Prism 7.0.

## 3 RESULTS

### 3.1 Clinical signs

#### 3.1.1 Clinical signs post‐vaccination

Two ferrets of thirty (one given V1 and one given V2 vaccine) developed a small lump at the site of injection on the day following vaccination, which remained for 2 days. Ferrets did not show any signs of pain or discomfort.

#### 3.1.2 Clinical signs post‐challenge

**FIGURE 2:**
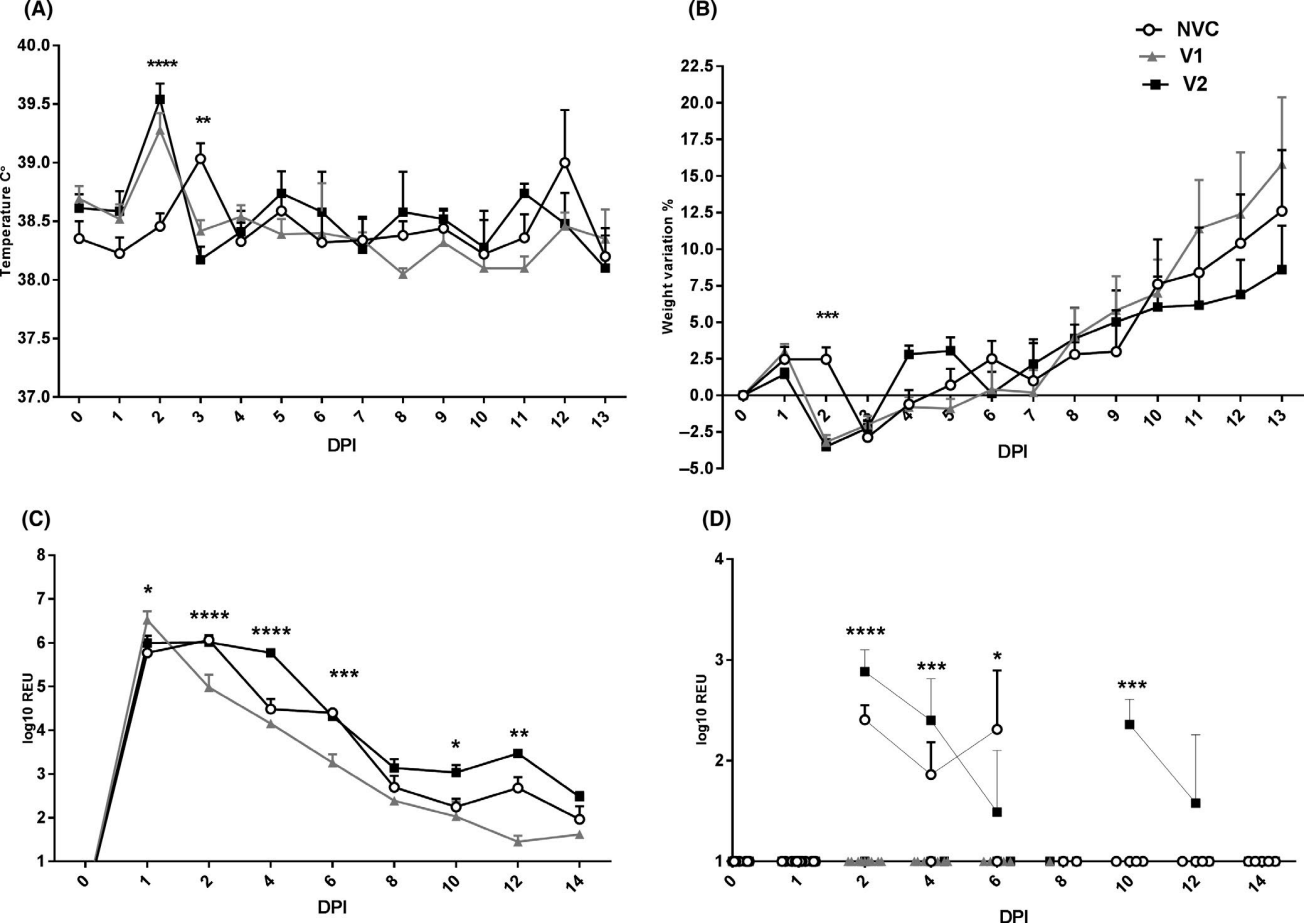
Clinical signs. Mean temperature (A) and weight (B) for each day post‐infection (DPI) are shown for groups V1 (V1 vaccine), V2 (V2 vaccine) and NVC (non‐vaccinated control). The NVC group showed significantly lower (*P* < .0001) and higher temperature (*P* < .01) than the vaccinated groups (V1 and V2) at 2 and 3 dpi, respectively. The NVC group showed significantly (*P* < .001) lower weight percentage in comparison with vaccinated groups (B), at 2 dpi. Viral RNA detection and quantification by RT‐qPCR in nasal washes (C). The V1 group showed significantly higher viral loads at 1 dpi in comparison with the V2 and NVC groups (*P* < .05) and significantly lower viral loads than the V2 and NVC groups at 2, 6 (*P* < .0001) and 12 dpi (*P* < .01). The V2 group showed significantly higher viral loads in comparison with both the NVC and the V1 groups at 4 dpi (*P* < .0001) than the V1 group at 10 dpi (*P* < .05). Viral RNA detection and quantification by RT‐qPCR in rectal swabs (D). V1 presented with significantly lower viral loads than the V2 and NVC groups at 2 (*P* < .0001), 4 (*P* < .001) and 6 (*P* < .05) dpi. The V2 group showed significantly higher viral loads than the NVC and the V1 groups at 10 dpi (*P* < .001). RT‐qPCR graphs show relative equivalent units (REUs). The assay limit of RNA detection is reached at an REU of 1. All samples below the assay limit are shown as 1. Mean ± standard error of the mean (SEM). Statistical analysis was carried out using two‐way analysis of variance. Differences were considered statistically significant at *P* < .05, *P* < .01, *P* < .001 and *P* < .0001, and represented as *, **, *** or ****, respectively

A significant transient elevation in temperature was observed in vaccinated animals on 2 dpi in comparison with the NVC group (*P* < .0001) and in NVC in comparison with vaccinated groups (*P* < .01), at 3 dpi (Figure 2A). The variation in body temperature was consistent with that observed for viral shedding level (Figure 2C).

Following infection, all infected ferrets lost over 2.5% of their total body weight (Figure 2A) at 1 dpi. The NVC group had a significant (P < .001) weight loss percentage in comparison with vaccinated groups (Figure 2B), at 2 dpi. The V2 group showed lower body weight in comparison with the V1 and NVC groups at 4 and 5 dpi. From 6 dpi, animals in all groups present a steady weight gain percentage.

### 3.2 Viral shedding

Nasal shedding data demonstrated that virus replication (RNA) was significantly higher in the V1 group in comparison with V2 (*P* < .05) and NVC (*P* < .001) at 1 dpi. In contrast, the V1 group showed significantly reduced viral RNA levels than the NVC and V2 groups at 2 (*P* < .0001) and 6 dpi (*P* < .001). At 12 dpi, the V1 group presented significantly reduced viral RNA levels than the NVC group  (*P* < .01) and the V2 group (*P* < .0001). Significantly higher viral RNA levels were detected in the nasal wash of the V2 group in comparison with the NVC and V1 groups at 4 dpi (*P* < .0001), and in comparison to the V1 group at 10 dpi (*P* < .05) (Figure 2C).

Low levels of rectal shedding (RNA) were detected in the NVC group and V2 groups but no RNA was detected in the V1 group following challenge. The overall mean rectal shedding in the V1 group was significantly reduced in comparison with the NVC and V2 groups at 2, 4 and 6 dpi (*P* < .05). In addition, significantly higher viral RNA levels were observed in the V2 group in comparison with the NVC and V1 groups at 10 dpi (*P* < .001) (Figure 2D).

### 3.3 Histopathology

**FIGURE 3:**
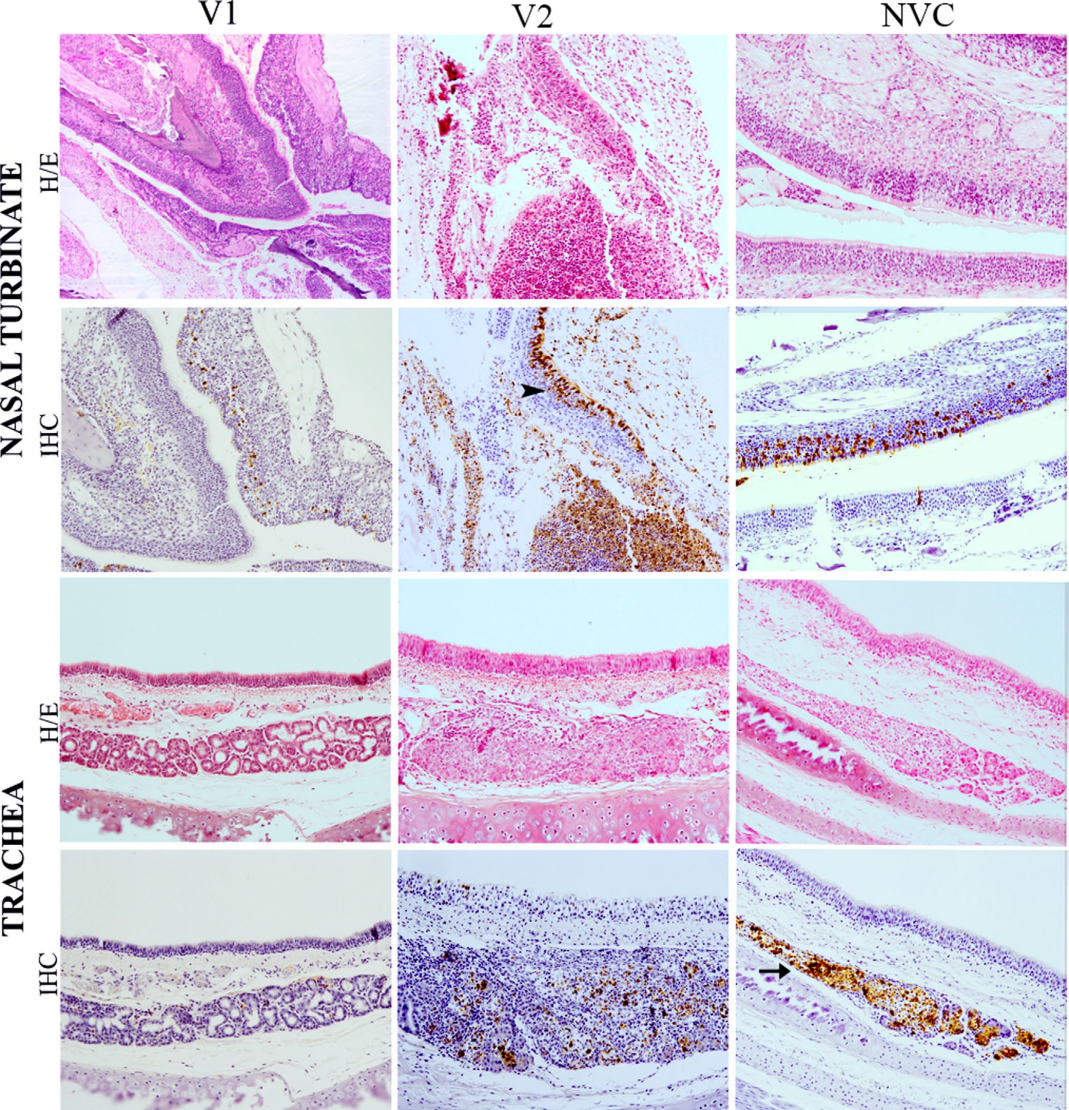
Histopathological lesions and immunohistochemical detection of influenza virus nucleoprotein in nasal turbinates and trachea at 5 dpi. Haematoxylin and eosin (HE) staining of tissues and immunohistochemical (IHC) detection of influenza virus nucleoprotein (NP) showed a moderate fibrinosuppurative rhinitis and lymphoplasmacytic tracheitis observed in group V2, characterised by increased number of inflammatory infiltrates and epithelial damage than the V1 (vaccine 1) group and the non‐vaccinated control (NVC) group. Viral NP immunolabelling observed in respiratory and olfactory epithelium (arrowhead) and glandular epithelium (arrow) of the trachea and nasal turbinates in all groups. Magnification 10x

Histopathological examination of the nasal turbinate and trachea, as well as, histopathological scoring of the lungs were performed at 3 and 5 dpi. NVC animals showed more severe histopathological lesions in the upper respiratory tract (URT) (nasal turbinate and trachea), than both vaccinated groups at 3 dpi. V1 group ferrets showed only mild lesions in nasal turbinate and trachea at 3 dpi, and no substantial lesions were observed at 5 dpi. In contrast, ferrets belonging to the V2 group showed slightly increased severity of lesions than the NVC and the V1 groups at 5 dpi. More substantial lesions observed in the nasal turbinate and trachea were consistent with a moderate fibrinosuppurative rhinitis and a moderate lymphoplasmacytic tracheitis, respectively (Figure 3).

**FIGURE 4:**
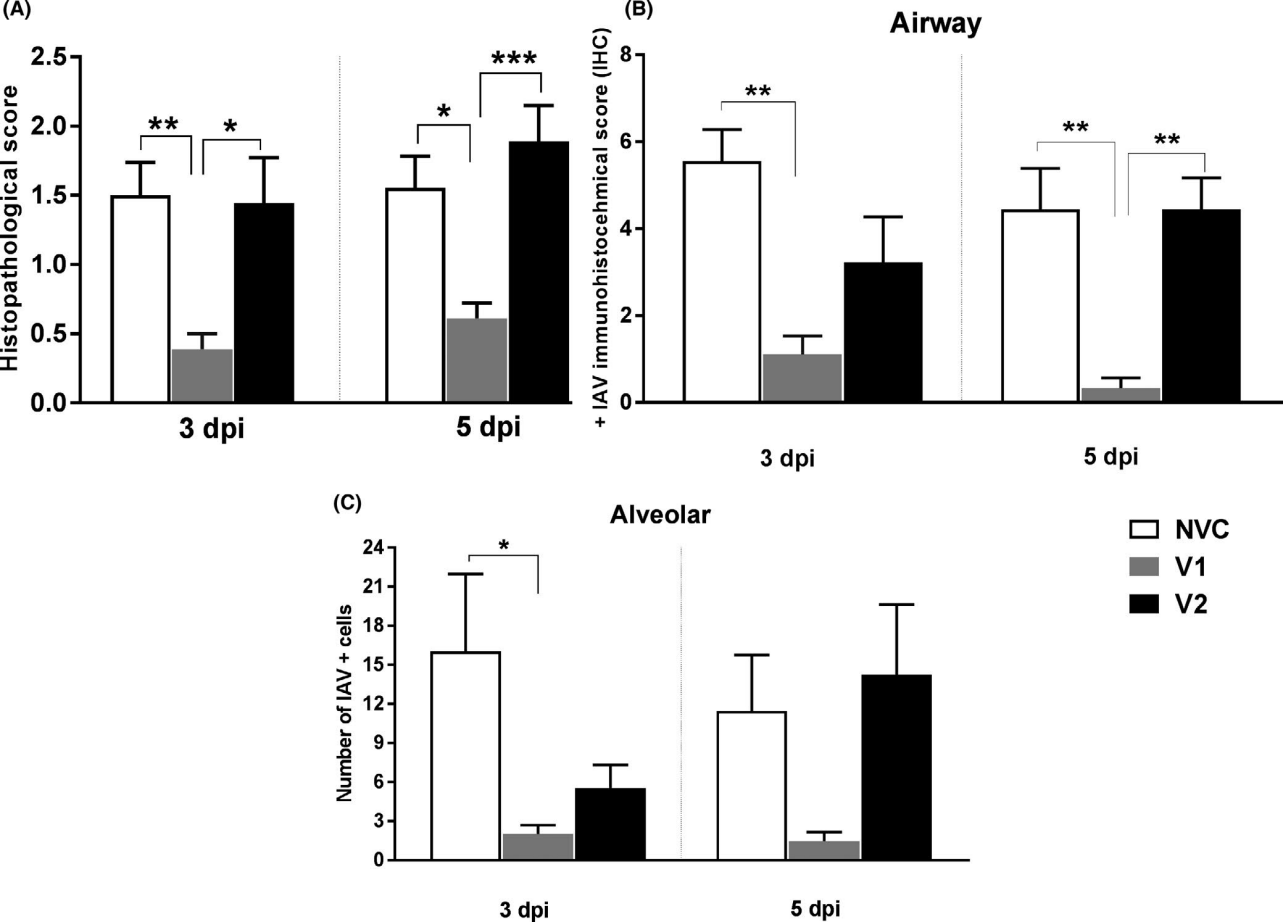
Pulmonary histological scores (A). Mean histopathological score of lung lobes. Mean ± SEM is shown. The V1 group showed significantly lower histopathological scores than the NVC (*P* < .01 and .05) and the V2 groups (*P* < .05 and .001), at 3 and 5 dpi, respectively. Influenza A virus antigen immunolabelling score in airway (bronchial/bronchiolar) (B) and alveolar (C) areas at 3 and 5 dpi. The V1 group had significantly lower nucleoprotein (NP) immunolabelling scores than the NVC group in airway (*P* < .01) and alveolar areas (*P* < .05) at 3 dpi. The V1 group also showed significantly lower immunolabelling scores than the NVC (*P* < .01) and the V2 (*P* < .01) groups in airway areas at 5 dpi. Mean ± SEM of NP‐positive immunolabelling per lung lobe. Statistical analysis was carried out using one‐way analysis of variance. Differences were considered statistically significant at *P* < .05, *P* < .01, *P* < .001 and *P* < .0001, and represented as *, **, *** or ****, respectively

**FIGURE 5:**
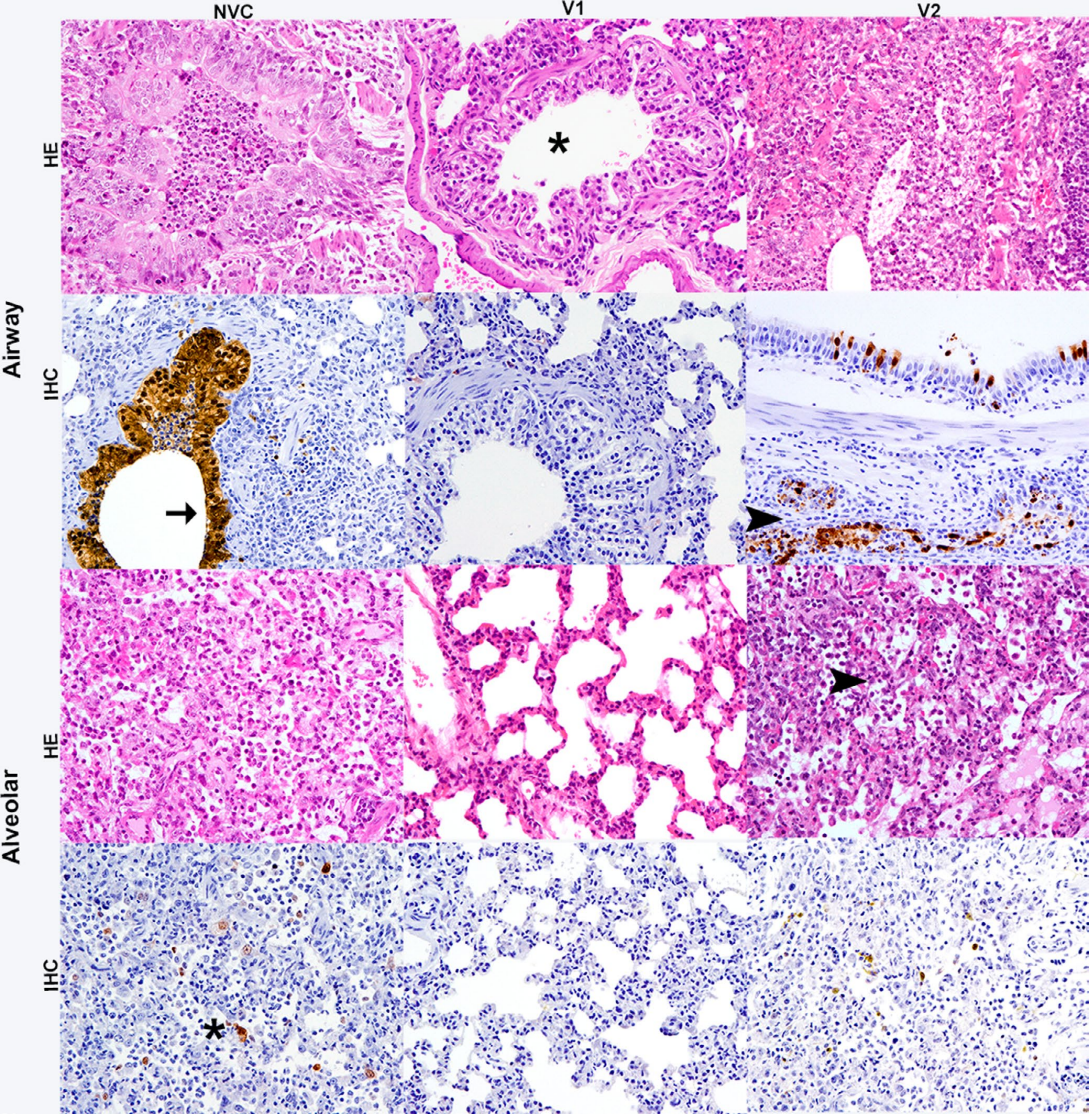
Lung histopathological lesions and viral antigen immunohistochemistry (IHC) in airway and alveolar areas at 3 days post‐infection (dpi). Haematoxylin and eosin (HE) stain revealed that lesions ranged from absent (star) to minimal bronchiolitis in the vaccine 1 (V1) group to more severe lesions observed in the vaccine 2 (V2) group and the non‐vaccinated control (NVC) group. Lesions in the V2 and NVC groups evaluated in HE stain were similar and were consistent with a mild to moderate bronchointerstitial pneumonia characterised by airway and alveolar epithelial necrosis accompanied by inflammatory infiltrates filling the alveolar spaces (arrowhead) and airways. Magnification 20×. Increased numbers of viral nucleoprotein immunolabelled cells were observed by IHC in the V2 and NVC groups in comparison with the V1 group. Positive immunolabelling by IHC was observed in the airway (arrow), glandular epithelium (arrowhead) and the alveolar epithelium (star) of NVC and V2 group ferrets. Magnification 20x

In the lungs, ferrets in the V1 group showed significantly (*P* < .05) lower histopathological scores at both 3 and 5 dpi when compared to the NVC group, and when compared to the V2 group (*P* < .05) (Figure 4A) at 5 dpi. These results mirrored the histopathology and IHC at 3 dpi, where no or minimal histopathological lesions were observed in V1. Lesions were characterised by a minimal bronchiolitis (star) with the presence of no or minimal lymphocyte and macrophage infiltrates (Figure 5). NVC and V2 groups showed similar histopathological scores to each other at 3 dpi. Highest scores were observed at 5 dpi in V2‐vaccinated animals (Figure 4A), showing increased severity of lesions in comparison with the V1 and NVC groups. More substantial histopathological lesions in V2 were consistent with a moderate bronchointerstitial pneumonia. Lesions were characterised by variable bronchiolar and alveolar epithelial necrosis, lymphoplasmacytic infiltration, with mucus and cell debris filling the bronchioles (arrow) and/or the alveoli (arrowhead) (Figure 5). Two animals per time point (3 and 5 dpi) showed interstitial lung lesions in the V2 group, while only one ferret per time point showed the same lesions in the NVC group.

### 3.4 Viral antigen detection and quantification

Viral detection by IHC was performed in nasal turbinate, trachea and lung fixed tissues at 3 and 5 dpi. In general, viral antigen was observed in the nucleus and cytoplasm of respiratory and olfactory epithelial cells (arrowhead) and glandular cells (arrow) in the nasal turbinates and trachea (Figure 3). Stronger IAV IHC immunolabelling was observed in the nasal turbinates of the V2 group, followed by the NVC and V1 groups, at both 3 and 5 dpi. In the trachea, the NVC group showed the strongest IAV antigen immunolabelling, followed by the V2 group, at 5 dpi.

In the lungs, viral antigen was observed in bronchial/bronchiolar epithelial (arrow) and glandular cells (arrowhead), alveolar epithelial cells (star) and few macrophages of animals belonging to the V2 and NVC groups (Figure 5). Semi‐quantitative evaluation of influenza virus nucleoprotein‐positive cells in the lungs showed that tissues from the V1 group had significantly lower (*P* < .05) IHC‐positive scores in airway (bronchial/bronchiolar) (Figure 4B) and alveolar areas (Figure 4C) at 3 dpi in comparison with the NVC and V2 groups, but no differences were observed between the V2 and NVC groups. At 5 dpi, the V1 group presented significant lower positive scores in comparison with the V2 and NVC groups in the airways (bronchial/bronchiolar areas) (Figure 4B), while no significant differences were observed between groups in alveolar areas at this time point (Figure 4C).

### 3.5 Tissue viral load

**FIGURE 6:**
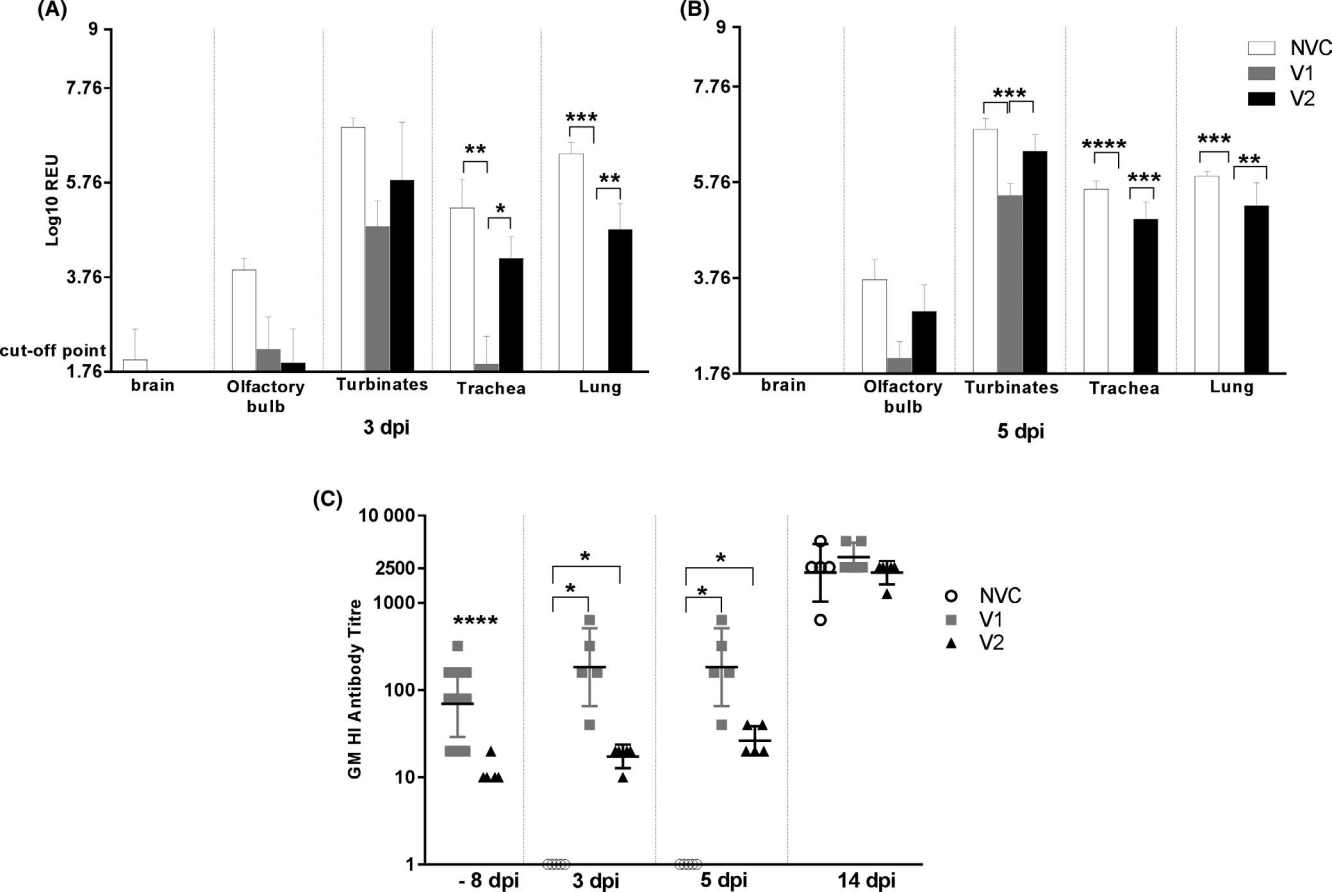
Tissue distribution of influenza virus at 3 dpi (A) and 5 dpi (B). Mean ± standard error of the mean (SEM). Viral RNA quantification by RT‐qPCR on tissue homogenates. The V1 group showed significantly lower viral loads than the NVC and V2 groups in the trachea (*P* < .01 and .05) and lung (*P* < .001 and .01) at 3 dpi. The V1 group showed significantly lower viral load than the NVC and V2 groups in nasal turbinates (*P* < .001), trachea (*P* < .0001(NVC) and *P* < .001(V2)) and lung (*P* < .001(NVC) and *P* < .01(V2)) at 5 dpi. Haemagglutination inhibition (HI) antibody titre (C). Pre‐ and post‐challenge geometric mean (GM) HI antibody response against A/Eng virus. Geometric mean and geometric standard deviation (SD). The V1 vaccine showed significantly higher HI titres than the V2 group at −8 dpi (*P* < .0001) and, than both the V2 and the NVC groups at 3 (*P* < .05 and 5 dpi (*P* < .05). Statistical analysis was carried out using Student's *t* test analysis for testing the differences between HI titres at −8 dpi/34 dpv between vaccinated groups and one‐way analysis of variance for the rest of all variables at each time point post‐infection. Differences were considered statistically significant at *P* < .05, *P* < .01, *P* < .001 and *P* < .0001, and represented as *, **, *** or ****, respectively.

Following challenge, brain, olfactory bulb, nasal turbinate, trachea and lung were collected at 3 and 5 dpi (Figure 6A and B) and viral load was assessed by viral RNA quantification by RT‐qPCR. At 3 dpi, no detectable viral RNA levels were detected in the brain of animals belonging to the vaccinated groups. Olfactory bulb viral RNA levels were lower in vaccinated groups than in the NVC control group. At 5 dpi, all three groups showed similar negligible viral loads in the brain and V2 and NVC showed similar viral replication in the olfactory bulb, which were higher in comparison with the V1 group.

In thoracic tissues, the V1 group showed the lowest viral load of the three groups at both 3 and 5 dpi, especially in comparison with NVC. In general, the V2 vaccine showed lower viral loads than the NVC group at 3 dpi. However, at 5 dpi, V2 and NVC showed similar viral load levels in the upper and lower respiratory tract. In the V1 group, there was significant (*P* < .005) control of viral load in the turbinates, at 5 dpi, in comparison with the NVC group. In contrast, the V2 group showed similar viral loads to that of the NVC group at 5 dpi. The reduction of viral load by V1 was evident (*P* < .001) in both the URT (trachea and turbinates) and the lung. V2 had less effect than V1 but, nonetheless, was able to significantly reduce viral load (*P* < .001) in both the URT (NS) and the lung.

### 3.6 Serology

During immunisation, V1‐immunised ferrets showed HI antibody titres ranging from 16 up to 1024, while V2‐immunised ferrets and AS03 adjuvant only group presented with negative antibody titres of <8 over the same time period (1 week to 34 dpv) (Table 1). Micro‐neutralisation titres (MNT) were similar, for V1 ranged from 3.3 to 383.2, while V2 MNT titres were only detectable late post‐immunisation (DPV 28‐24) at 10‐13 titres (data not shown).

**Table 1:** Haemagglutination inhibition (HI) antibody titres of immunised ferrets during vaccination

Group	Days post‐immunisation								
	Ferret ID	0	7	11	14	18	21	28	34
							Boost		
V1 vaccine	1	0	16	128	256	256	32	32	128
	2	0	256	512	512	1024	512	512	512
	3	0	32	256	512	NS	128	128	256
V2 vaccine	1	0	<8	<8	<8	NS	<8	<8	<8
	2	0	<8	<8	<8	<8	<8	<8	<8
	3	0	<8	<8	<8	<8	<8	<8	<8
AS03 Adjuvant	1	0	<8	<8	<8	NS	NS	NS	<8
	2	0	<8	<8	<8	NS	<8	NS	<8
	3	0	<8	<8	<8	NS	<8	<8	NS
NVC	1	0	<8	<8	<8	NS	NS	NS	<8

Abbreviations: NS, no sample; NVC, non‐vaccinated control.

Following the immunisation, 8 days prior to challenge and 34 dpv, homologous antibodies were detected in the V1 group. These titres were significantly higher than those in the V2 group, which showed very low levels (*P* < .001) (Figure 6C). Post‐challenge, antibody levels against A/Eng increased at 3 and 5 dpi, with V1 titres remaining significantly higher than V2 titres (*P* < .005). No antibodies were detected at 3 or 5 dpi in the NVC group. At 14 dpi, high levels of homologous antibodies were detected in all groups (Figure 6C), with no statistically significant differences were detected between groups. The anamnestic response in the V1 group saw a ~50‐fold increase in titres from 22 days post‐vaccine to 14 days post‐challenge, whereas for V2 this was ~500‐fold increase; both vaccines attained approximately the same final titre at 14 dpi (Figure 6C).

## 4 DISCUSSION

Human vaccines tested in this study using the ferret model showed differences in their efficacy against diverse parameters of A(H1N1)pdm09 infection resulting in different infection outcomes. The adjuvanted inactivated split virion vaccine (V1) limited and controlled viral shedding and replication outside the airway and clearly reduced respiratory tract infection. In contrast, the non‐adjuvanted inactivated whole virion vaccine (V2) appeared to delay respiratory tract infection with a slight reduction of viral shedding but resulting in similar lung lesions in comparison with the NVC group and much less marked effect on virus replication in tissues outside the respiratory tract.

The HI assay is commonly used to measure anti‐HA antibodies for vaccine evaluations, and HI antibody titres ≥ 40 have traditionally been considered to be protective against infection with a homologous influenza A strain.^14^, ^15^ In this study, the immunogenicity of the two vaccines was significantly different. A single dose of 3.75 µg AS03B adjuvanted subunit vaccine (V1) was more immunogenic than two doses of 7.5 µg whole virus vaccine (V2) as per vaccine dosing guidelines in humans. In the V2 group, vaccination did not elicit a high anti‐HA antibody response during the 34 days post‐immunisation, while the V1 group produced detectable titres from 1 week post‐immunisation onwards; results were similar for the immunisation and challenge study, and for virus neutralisation assays. The observed differences in vaccine immunogenicity in the ferret model are consistent with the observed superiority of the immune response to V1 vaccines in paediatric head‐to‐head comparison clinical studies in the UK.^6^

Previous studies have shown that the presence of HI antibody titres is often low at early stages post‐infection in individuals previously vaccinated with A(H1N1)pdm09 vaccine.^16^ However, the presence of cross‐reactive antibodies and the secondary antibody response from memory B cells play an important role in the control of viral replication during this early phase of infection. In this study, vaccines tested provided different protection levels against high‐dose virus challenge in the naive ferret model. Differences between the vaccines were noted using clinical, pathological and virological end points, where the adjuvanted inactivated vaccine V1 showed higher efficacy and better protection than the non‐adjuvanted V2 vaccine.

A substantial antibody response was seen following virus challenge in animals immunised with V1 vaccine between days 3 and 14, whereas this was not observed until day 8‐14 post‐challenge in ferrets immunised with the V2 vaccine. In addition, the V2 group showed higher temperature after 8 dpi in comparison with the V1 group that was associated with higher viral shedding from the rectal and nasal swabs at the same time points, and more severe lung pathology.

The V1 group showed limited spread of viral antigen in the lungs consistent with the minimal to mild histopathological pulmonary lesions observed. These results confirm the higher level of protection achieved by the V1 vaccine against viral replication in comparison with other inactivated vaccines, as reported previously.^17^, ^18^, ^19^ V2 and NVC showed similar histopathology in lungs following challenge. These lesions were characterised by a moderate lymphoplasmacytic bronchointerstitial pneumonia that was associated with the presence of higher IAV antigen counts in alveolar areas. These results are in accordance with previous studies where more severe respiratory outcomes after A(H1N1)pdm09 infection in ferrets and humans were related to the spread of viral antigen to alveolar areas and the associated inflammatory response.^10^, ^20^

At 5 dpi, the V2 group showed similar to slightly increased severity on lung histopathological lesions compared to the NVC group, in accordance with the presence of similar RNA viral levels in the lungs and other organs between these groups. These results may indicate that viral replication was only delayed but not controlled by V2 vaccine, resulting in lung pathology. It is known that susceptibility to IAV‐associated lung pathology in humans is partially determined by multiple host factor characteristics, which include prior immunity, co‐morbidities and host genetic characteristics.^21^, ^22^ Consequently, basal immune alterations, and/or the presence of a previous inflammatory state, may impact upon the normal development of specific immune response against A(H1N1)pdm09, and increase the risk of developing poor outcome of infection or antibody adverse immune reactions. Therefore, the use of vaccines which modulate but do not control/prevent infection may pose a substantial health threat for their use in higher risk individuals.

Non‐neutralising antibodies against IAV can have either protective, neutral non‐protective or detrimental effects.^23^ Detrimental effects produced by non‐neutralising antibodies have been suggested to be related to poor outcomes of various viruses, including A(H1N1)pdm09.^24^, ^25^ In addition, a delay in viral clearance rather than a high viral load in the respiratory tract has been associated with severe disease in human patients, which may reflect the delay of infection parameters observed in the V2 group in this study. ^23^

V2 group lung lesions at 5 dpi were mostly associated with viral antigen presence in alveolar areas, a consequence that is most likely related to poor induction of specific A(H1N1)pdm09 antibodies during immunisation in this group and to the detrimental inflammatory cellular responses as described elsewhere.^10^, ^26^, ^27^ However, the V2 group showed some antibody response during infection, that was higher than the non‐vaccinated challenge group, and lower viral antigen presence than the NVC control group by IHC, indicating some level of priming of the antibody response induced by the non‐adjuvanted vaccine.

In conclusion, in the ferret model of human influenza, the AS03 adjuvanted inactivated vaccine V1 provided better protection against clinical disease following A(H1N1)pdm09 infection than the non‐adjuvanted inactivated V2 vaccine, which had a lower antibody response, higher viral load and clinical disease comparable to non‐vaccinated ferrets. These results identify the need for improved knowledge of head‐to‐head comparisons of vaccine performance in humans at the population level, and the usefulness of biological model systems for teasing out aspects of adaptive immune responses assisting with potential improvements of vaccine design and delivery.

## CONFLICT OF INTEREST

All authors declare that they have no conflict of interest.

## AUTHOR CONTRIBUTION

**Beatriz Vidana:** Data curation (equal); Formal analysis (equal); Investigation (equal); Methodology (equal); Software (lead); Visualization (lead); Writing‐original draft (lead); Writing‐review & editing (lead). **Sharon M. Brookes:** Funding acquisition (equal); Investigation (equal); Methodology (equal); Project administration (equal); Supervision (lead); Validation (equal); Writing‐original draft (equal); Writing‐review & editing (lead). **Helen Everett:** Data curation (equal); Formal analysis (supporting); Methodology (supporting); Supervision (equal); Writing‐original draft (supporting); Writing‐review & editing (supporting). **Fanny Allen:** Formal analysis (equal); Investigation (equal); Resources (equal); Writing‐review & editing (supporting). **Alejandro Nuñez:** Investigation (supporting); Methodology (equal); Supervision (supporting); Writing‐original draft (supporting); Writing‐review & editing (supporting). **Othmar G Engelhardt:** Data curation (supporting); Formal analysis (supporting); Supervision (supporting); Validation (supporting); Writing‐original draft (supporting); Writing‐review & editing (supporting). **Diane Major:** Resources (supporting); Supervision (supporting); Writing‐review & editing (supporting). **katja hoschler:** Formal analysis (supporting); Methodology (supporting); Resources (supporting); Writing‐review & editing (supporting). **Ian Brown:** Formal analysis (supporting); Project administration (supporting); Resources (supporting); Supervision (supporting); Validation (supporting); Writing‐review & editing (supporting). **Maria Zambon:** Conceptualization (lead); Data curation (equal); Formal analysis (lead); Funding acquisition (lead); Project administration (lead); Resources (equal); Supervision (lead); Writing‐original draft (equal); Writing‐review & editing (lead).
